# 

**Published:** 1887-04

**Authors:** 


### BOOK NOTICES.

The Open Court.— We extend the hand of fellowship, with a hearty God speed to *The Open Court*, a new bi-monthly, published in Chicago, under the conduct of B. F. and Sarah A. Underhill.

We like it, and all it says in a salutatory that has the ring of *true metal.*

Yes, *Open Court* there is not only *room* for you in the great procession of untrammelled thinkers, but there is *need* of you, as a color-bearer, and we hope to be able to distinguish your colors in the clear light of your own choosing for many a day.

Horticultural Art Journal.— We are glad to notice this exchange, both on account of its fine typographical appearance and its reading matter, which embraces much valuable information. It is handsomely illustrated with five colored plates. Published at Rochester, N. Y. Price, $3.00 per year.

Shoppell's Modern Houses.—We have received No 5 of this valuable quarterly publication, containing forty-nine designs for modern dwelling houses, with plans, descriptions and cost, varying from $1,000 to $12,000, besides other building designs, and valuable information, relative to the interior home and its surroundings.

Published at one dollar, by the co-operative Building Plan Association, 191 Broadway.

We are informed that 8,000 houses have been built from Shoppell's Plans, and he has as many more in his head. Send for the work by mail, if you would save money in building, and have a handsome home to live in.

Pocket Medical Formulary, arranged therapeutically, for the use of "the busy and hard-worked practitioner," with blank leaves for ready minutes or diagnoses, has been laid upon our table for inspection.

The work is a veritable *multum in parvo*, containing 334 pages. Eleven hundred formulas gathered of upwards of four hundred authorities, and will supply a long-felt want, which physicians in active practice are now able to supply at the very moderate cost of $2.00, on application to the Medical Formulary Publishing Company, 1208 Chestnut Street, Philadelphia.

